# A young female with seropositive rheumatoid arthritis complicated by cavitary rheumatoid nodules in the lung: Case report

**DOI:** 10.1097/MD.0000000000048485

**Published:** 2026-05-01

**Authors:** Weiran Li, Sai Yuan, Xue Wu, Zhongping Wang, Jin Guo, Hao Yang, Mao Hua

**Affiliations:** a The Affiliated Hospital of Qingdao University, Qingdao, Shandong Province, China; b Clinical Medical College of Qinghai University, Xining, Qinghai Province, China; c Binhai County Hospital of Traditional Chinese Medicine, Yancheng, Jiangsu Province, China; d The 88th Hospital of the People’s Liberation Army of China, Taian, Shandong Province, China.

**Keywords:** case report, cavitation, pulmonary involvement, pulmonary rheumatoid nodules, rheumatoid arthritis

## Abstract

**Rationale:**

Rheumatoid arthritis (RA) is a systemic autoimmune disease characterized by inflammatory arthritis and extra-articular involvement, with lung involvement being the most common extra-articular manifestation. Rheumatoid nodules are highly specific for RA, occurring in 20% of patients and are more common in male patients with a history of smoking. This article aims to explore the diagnostic approach, key points of differential diagnosis, and treatment strategies of this disease by reporting the diagnosis and treatment process of a case of RA complicated with rheumatoid nodules in the lungs with cavity formation, and by reviewing the literature, providing diagnostic ideas for clinicians in treating similar cases.

**Patient concerns:**

The patient had a history of a persistent cough of unknown etiology, which persisted despite medical therapy. Additionally, the patient developed symmetric, progressive polyarticular pain, a key manifestation of the underlying disease.

**Diagnoses:**

This case was diagnosed as rheumatoid nodules in the lungs through a comprehensive analysis of the patient’s clinical history (including RA and related symptoms), laboratory tests (elevated inflammatory markers and positive specific antibodies), chest CT imaging features (multiple nodules with cavities in both lungs), and pathological examination results (excluding tumors, tuberculosis and fungal infections, showing fibrinoid necrosis), and based on a multidisciplinary consultation.

**Interventions:**

The patient received comprehensive treatment including hormones and antirheumatic drugs to relieve symptoms and control the progression of RA.

**Outcomes:**

One year after discharge, the patient’s pulmonary lesions gradually subsided and there was no disease progression.

**Lessons:**

For RA patients with positive serology, if multiple subpleural nodules or masses with cavities are found on chest CT, pulmonary rheumatoid nodules should be considered first. However, tuberculosis, fungal infection, tumor, and vasculitis must be systematically excluded. The pathology may be atypical, and the consistency of clinical, imaging and pathological findings and dynamic follow-up should be emphasized.

## 1. Introduction

Rheumatoid arthritis (RA) is a systemic autoimmune disease, characterized by symmetrical, chronic progressive inflammatory arthritis, and may involve multiple extra-articular systems. According to epidemiological data, the global prevalence of RA is ~0.24%.^[1]^ RA is more common in women than in men, with a lifetime risk of 3.6% in women and 1.7% in men.^[2]^ The lungs are one of the most frequently involved extra-articular organs in RA, and its pulmonary manifestations present a “spectrum” distribution, including RA-related interstitial lung disease, rheumatoid nodules in the lungs, airway lesions (such as bronchiolitis/bronchiectasis), pleural involvement (pleural effusion/pleural thickening), pulmonary vasculitis, and drug-related lung injury, etc.

Pulmonary rheumatoid nodules are relatively rare pulmonary manifestations of RA, often located in the periphery of the lungs and subpleural areas, which can be single or multiple. Some may undergo necrosis and form cavities, thus being highly similar to pulmonary tuberculosis, fungal infections, granulomatous diseases, and lung malignancies on imaging, leading to misdiagnosis or delayed diagnosis. Therefore, the diagnosis of this type of lesion usually relies on consistent evidence from “clinical - imaging - pathological” aspects and requires systematic exclusion of more common causes such as infection and tumors. In necessary cases, repeated sampling and multidisciplinary discussions are of crucial value.

This article reports a case of a young female RA patient with positive serology, presenting with multiple subpleural nodules and masses with cavities in both lungs on imaging. The diagnosis of this case involved multiple samplings, microbiological examinations, and multidisciplinary consultations, and was ultimately diagnosed as pulmonary rheumatoid nodules. Follow-up imaging showed that the lesions had certain dynamic changes and gradually absorbed after immunotherapy. This article aims to summarize the diagnostic reasoning and key points of differential diagnosis of this case, in order to provide a clearer approach for the clinical management of similar “cavitary pulmonary nodules.”

## 2. Case report

### 2.1. Methodology/ethics

This study was approved by the Ethics Committee of the Medical College of Qinghai University. Patients have signed written informed consent, agreeing to the publication of relevant clinical data and images. This case report was written in accordance with the CARE guidelines.

### 2.2. Case background

The patient is a 33-year-old female. She visited our hospital on July 1, 2024, due to joint pain for 10 months, which worsened with coughing for 3 months. Ten months ago, she developed symmetrical pain in the toe and knee joints without any obvious cause. The pain was initially mild but gradually spread to both wrist and metacarpophalangeal joints. There was no significant morning stiffness, and no long-term fever or night sweats indicative of tuberculosis intoxication symptoms. She did not pay much attention to it or receive any treatment. Three months ago, due to severe joint pain that prevented her from moving, she was diagnosed with “rheumatoid arthritis” at a local hospital. A plain chest CT scan revealed multiple irregular soft tissue nodules in both lungs, and multiple enlarged lymph nodes in the mediastinum and bilateral axillae. Enhanced chest CT showed multiple irregular soft tissue nodules under the pleura in both lungs. Color Doppler ultrasound of superficial lymph nodes showed enlarged lymph nodes in both axillae, the left inguinal region, and the left supraclavicular area. A CT-guided lung biopsy of the left lung indicated chronic inflammation of fibrous connective tissue, with focal mesothelial coverage, and numerous lymphocytes, histiocytes infiltration, hemorrhage, and edema beneath it. On April 8, 2024, she was prescribed leflunomide (LEF) tablets (25mg twice daily), methotrexate tablets (10 mg once a week), and celecoxib for RA. After discharge, her joint pain persisted, and she gradually developed intermittent coughing, which was more pronounced when lying on her left side, with a small amount of white foamy sputum.

### 2.3. Clinical evaluation

Physical examination: SPO2: 89% (without oxygen inhalation), rechecked and recovered to 92% to 98% after treatment (with 2 L/min oxygen inhalation), no persistent hypoxia occurred. Enlarged lymph nodes were palpable in the supraclavicular and axillary regions. Coarse breath sounds were heard in both lungs on auscultation, and a few moist rales were heard in the right lower lung. Specialized examination: Pain in the proximal interphalangeal joints, metacarpophalangeal joints, wrist joints, and bilateral knee joints, with mild limitation of limb movement. No joint redness, swelling, or deformity was observed. Laboratory and imaging tests: on July 4, 2024, rheumatism: C-reactive protein, 73.50 mg/L; antistreptolysin O, 139.0 IU/mL; rheumatoid factor (RF), 309.0 IU/mL; anti-keratin antibody: (+); anti-cyclic citrullinated peptide antibody (CCP): 85.53 RU/mL; d-dimer, 7.1 mg/L; erythrocyte sedimentation rate, 65.00 mm/h; no significant abnormalities were found in the blood routine; blood ANA spectrum, ANCA, G/GM test, echinococcus IgG antibody, tumor markers, TB-DNA, T-SPOT, acid-fast staining, IGRA, ANCA, general bacteria and fungi culture, and direct fungal smear were all negative. Chest CT: Multiple mixed-density masses in both lungs, with scattered mildly enlarged lymph nodes in the mediastinum. No significant abnormalities were found in the anteroposterior x-ray of both hands and the anteroposterior and lateral x-rays of both knees. Superficial lymph node color Doppler ultrasound: Enlarged left supraclavicular lymph node (19 mm × 5 mm), enlarged bilateral axillary lymph nodes (15 mm × 10 mm), and enlarged bilateral inguinal lymph nodes. No abnormalities were found in gynecological color Doppler ultrasound and color Doppler ultrasound of both lower extremity deep veins. Due to the patient’s personal reasons, blood gas analysis, pulmonary function, and PET-CT examinations were refused.

Pathological examination and diagnostic process (summary of diagnostic reasoning is shown in Table 1): Firstly, the lung tissue slice from another hospital was reexamined and showed: (left lung) no alveolar epithelium was seen in the fibro-fatty tissue, only a small amount of mesothelium was present, and lymphocytes and many focal histiocytes were visible. To clarify the nature of the lung mass, ultrasound-guided lung biopsy was performed again. At the same time, endobronchial ultrasound was conducted, and mediastinal lymph node puncture and BALF NGS were sent for examination. NGS did not detect pathogenic bacterial sequences, and the pathological result of mediastinal lymph node biopsy (pathological results are shown in Table 2) was obtained.

**Table 1:** Diagnostic reasoning summary.

Diagnose problems	Evidence in this case (from medical history/examination)	Counter-evidence/insufficiency	Inference conclusion (supportive statement)
How to confirm RA	(1) Clinical manifestations: Symmetrical polyarticular pain lasting for 10 mo, involving toe joints, knee joints, wrist joints, and metacarpophalangeal joints; the duration exceeded 6 wk. (2) Inflammation: Elevated ESR at 65 mm/h and CRP at 73.5 mg/L. (3) Serology: elevated RF at 309 IU/mL; positive anti-CCP at 85.53 RU/mL; positive anti-keratin antibody	The patient has no obvious morning stiffness. The imaging (x-ray of hands/knees) shows no bone erosion, which does not affect the classification, but suggests that the disease may still be in the early stage	All the key elements required for the 2010 ACR/EULAR classification (joint involvement + serology + acute phase reactants + disease duration) are present in this case. Therefore, the diagnosis of RA in this case is supported by sufficient clinical and serological evidence
How to rule out infection (TB/fungi mainly)	(1) Tuberculosis: All tests including TB-DNA, T-SPOT, IGRA, and acid-fast staining were negative; no pathogenic bacterial sequences were detected in BALF NGS. (2) Fungi: Fungal culture, direct smear, and blood G/GM test were negative; no fungal sequences were detected in NGS; no morphological evidence of fungi was found in pathology. (3) Clinical manifestations: No long-term fever or night sweats, or other symptoms of tuberculosis intoxication	All tests including blood G/GM, fungal culture, direct fungal smear, TB-DNA, T-SPOT, IGRA, and acid-fast staining were negative. However, dynamic reexamination is still required	In the context of repeated cultures, negative histological sampling with acid-fast staining, and no pathogenic evidence detected by NGS of BALF, along with the absence of typical infection symptoms, there is insufficient evidence to support an infectious nodule (tuberculosis/fungal), but follow-up is still necessary to be vigilant for secondary infection in the cavity
How to rule out tumors	No clear evidence of tumor was found in multiple samplings: lung biopsy indicated acute and chronic inflammatory cell infiltration, necrotic tissue, and histiocytes (Table 2); mediastinal lymph node puncture showed mainly lymphoid tissue/lymphocytes (Table 2), with no description of atypical cells or obvious mitotic figures; all tumor markers were normal (2.2). Imaging: Enhanced CT showed no central enhancement of the lesion, with peripheral enhancement, consistent with one of the imaging features of necrotic nodules/granulomatous lesions (Table 3)	Negative tumor markers cannot rule out tumors; there is sampling error in puncture, especially for necrotic tumors	Based on multiple pathological examinations that have not found evidence of tumor cells and the imaging results that are more consistent with the manifestations of necrotic/inflammatory nodules, there is insufficient evidence of malignancy in this case. It is still recommended to follow up with dynamic imaging monitoring as a safety measure
Why support pulmonary rheumatoid nodules (chain of supportive evidence)	(1) Background: RA patients with positive serology and significantly elevated inflammation. (2) Imaging: Multiple nodules/clusters in both lungs, mainly subpleural/peripheral, some with cavities/air, and dynamic fluctuations (waxing-waning) during follow-up (Table 3, Fig. 1). (3) Pathology: Necrotic tissue/fibrinoid necrosis, surrounded by histiocyte infiltration, CD68 (+), acid-fast staining (−; Table 2). (4) Treatment responsed imaging gradually improved af: Symptoms an ter treatment with glucocorticoids combined with DMARDs (Table 3, Fig. 1)	No typical “fence-like” arrangement of histiocytes was obtained, suggesting limited sampling/atypical morphology; the therapeutic response cannot be the sole basis for diagnosis and can only serve as supporting evidence	While excluding infection and tumor, this case has a consistent chain of evidence including “RA background + typical distribution imaging (multiple subpleural nodules with possible cavities) + necrotizing/histiocytic predominant pathology + follow-up dynamics and immunosuppressive response,” making the diagnosis of rheumatoid nodules with cavity formation in the lung the most reasonable

DMARD = disease-modifying antirheumatic drug, RA = rheumatoid arthritis.

**Table 2:** Pathological results.

Check type	Pathological results
April 2024: CT-guided lung puncture of the left lung at an outside hospital	Chronic inflammation of fibrous connective tissue is observed, with focal mesothelial covering. Underneath, there is a significant infiltration of lymphocytes and histiocytes, along with hemorrhage and edema
July 5, 2024: Resubmission of lung tissue slices from an outside hospital for examination	In the fibro-fatty tissue of the left lung, no alveolar epithelium was observed. Only a small amount of mesothelium was seen, along with lymphocytes and numerous foci of histiocytes
July 9, 2024: Ultrasound-guided lung puncture biopsy	The submitted puncture tissue shows patchy infiltration of acute and chronic inflammatory cells, with occasional histiocytes, fibrous tissue hyperplasia, partial fibrous tissue degeneration, mild hyperplasia, dilation and congestion of small blood vessel endothelial cells. Small areas of necrotic tissue are also observed, surrounded by a few epithelioid cells and a small amount of striated muscle tissue. Immunohistochemical results: CD68 positive for histiocytes. Special staining results: acid-fast staining negative
July 16, 2024: Mediastinal lymph node puncture(7th group lymph node biopsy)	Under microscopic examination, the coagulated tissue shows clusters of lymphocytes with fibrinoid necrosis
July 16, 2024: Liquid-based cytology of bronchoalveolar lavage fluid	Moderate neutrophils, a few ciliated columnar epithelial cells
July 16, 2024: Liquid-based cytology of lung brushings (right lower basal segment)	A small amount of ciliated columnar epithelium
July 18, 2024: Pathology of fiberoptic bronchoscopy (right lower basal segment lesion)	Microscopic examination of the tissue revealed ciliated columnar epithelium lining. In the stroma, thick-walled blood vessels, a few smooth muscle bundles and collapsed lung tissue were observed. The thick-walled blood vessels were distributed in small foci, surrounded by compressed lymphocytes. A small amount of fragmented ciliated columnar epithelium was also seen

Follow-up CT showed that the size of the nodules and the formation of cavities fluctuated asynchronously, but the overall trend was gradually reduced and absorbed after immunosuppressive treatment, which was consistent with the waxing-waning characteristics of rheumatoid nodules in the lung.^[3]^ The final diagnosis was established based on the consistency of clinical, imaging and pathological findings; given the limitations of biopsy sampling, long-term follow-up is still needed to further consolidate the diagnosis and monitor complications.

Final diagnosis: RA and rheumatoid nodules in the lung with cavity formation.

### 2.4. Treatment response and outcomes

Treatment interventions: After admission, the patient was given diclofenac sodium and lidocaine 20 mg intramuscular injection and celecoxib for pain relief, methotrexate 10 mg once a week, and LEF 25 mg twice a day for RA treatment, as well as acetylcysteine nebulization for bronchodilation and bromhexine for expectoration. After a clear diagnosis was made, the treatment plan was adjusted to prednisone 30mg once a day, methotrexate 10 mg once a week, and LEF 25 mg twice a day for RA treatment. Calcium supplementation and gastric mucosa protection were also provided to reduce the risk of osteoporosis and gastrointestinal adverse reactions.

**Figure 1.**
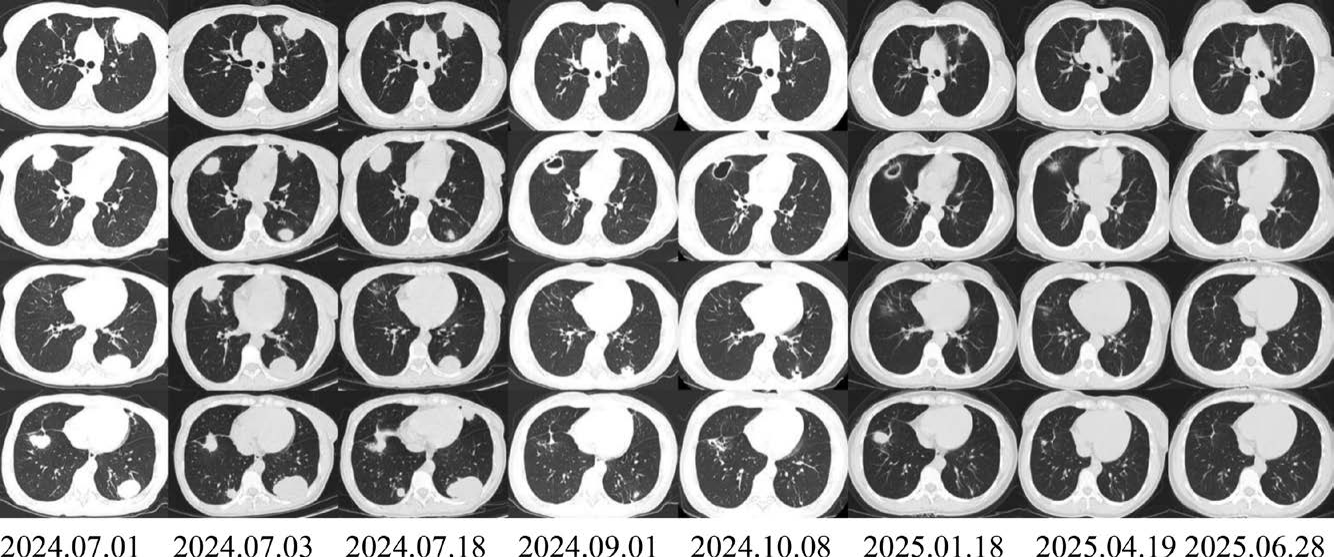
High-resolution CT of the patient’s lungs at the early stage of the disease. Multiple nodules of varying sizes can be seen in both lungs, some of which are round or nearly round, while others are irregular, with mixed density, mainly low density, and have shallow lobulation and spiculation. Some of them are fused. With the progress of treatment, the multiple mixed-density nodules and nodules in both lungs have significantly decreased in size and number. No cavities are shown in the lesions. Currently, multiple solid patchy and fibrous cord-like shadows are prominent in the local areas. CT = computed tomography.

**Figure 2.**
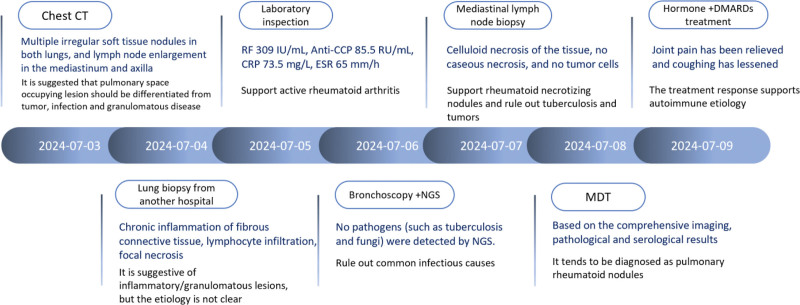
Patient diagnosis and treatment process: detailed steps from symptom discovery to confirmation of pulmonary rheumatoid nodules.

Treatment outcomes and follow-up: after treatment, the patient’s joint pain and cough symptoms were significantly relieved, and the condition was stable. The patient was discharged. One-year follow-up after discharge showed that the patient continued to receive standardized treatment, the condition remained stable, and there was no recurrence. During the follow-up period, after assessment by a rheumatology and immunology specialist, the patient discontinued methotrexate (MTX) on January 18, 2025, and was switched to LEF 20 mg/d po combined with tocilizumab 8 mg/kg ivgtt q4w. Prednisone was gradually reduced to 10mg/d for maintenance. The joint symptoms remained stable. A reexamination of the chest CT (see Table 3) showed that the pulmonary nodules and cavities had significantly absorbed and regressed compared to before. (To more clearly present the time correlation between drug exposure and the evolution of lung images, we have summarized the patient’s disease course timeline in Table 4, and the chest CT images for each time period are shown in Fig. 1. The process from the discovery of symptoms to the diagnosis of pulmonary rheumatoid nodules in the patient is shown in Fig. 2).

**Table 3:** Chest CT results and imaging descriptions at different time periods.

Time	Imaging description
April 2024 (outside the hospital)	Chest CT plain scan + enhanced scan: Pulmonary window shows: Multiple irregular soft tissue nodules are found in the subpleural areas of both lungs. Mediastinal window: Multiple lymph nodes in the mediastinum and bilateral axillae are partially enlarged
July 1, 2024 (two days before admission)	Chest CT plain scan: Pulmonary window shows multiple mixed density masses of varying sizes in both lungs, some are round or nearly round, and some have slightly irregular shapes, with low density being prominent. The margins of the lesions are slightly lobulated and spiculated, and some lesions are fused. The largest one is located in the posterior basal segment of the left lower lobe, measuring ~5.9 cm × 2.6 cm, with CT values ranging from 23.5 to 47.8 HU. Some bronchial shadows are visible within the lesions, and the boundaries are relatively clear. There is a cystic lucency of about 0.8 cm in the lateral segment of the middle lobe of the right lung. There are a few linear shadows in the middle lobe of the right lung Mediastinal window shows scattered mildly enlarged lymph nodes in the mediastinum; thickening of the lower dorsal pleura on both sides. Conclusion: Multiple mixed density masses of varying sizes in both lungs; emphysema in the lateral segment of the middle lobe of the right lung; a few linear shadows in the middle lobe of the right lung; thickening of the lower dorsal pleura on both sides. Scattered mildly enlarged lymph nodes in the mediastinum
July 3, 2024 (the day of admission)	Chest CT plain scan + enhanced scan: Pulmonary window shows: Multiple mixed-density masses in both lungs, with morphology and edge features similar to the previous examination (still showing shallow lobulation, spiculation and partial fusion), the largest one located in the posterior-basal segment of the left lower lobe, ~5.9 cm × 3.1 cm in size, CT value about 22.5–42.6 HU, some lesions have bronchial shadows passing through, with relatively clear boundaries. Enhanced scan shows no central enhancement, but obvious peripheral enhancement. A cystic lucency about 0.9 cm in the lateral segment of the right middle lobe; a few streaky shadows in the right middle lobe. Mediastinal window shows: Scattered mildly enlarged lymph nodes in the mediastinum; thickening of the lower dorsal pleura on both sides. Conclusion: Compared with the film on 2024-07-01: Multiple mixed-density masses in both lungs, with significant necrosis in the lesions; other manifestations are similar to the previous examination
July 18, 2024 (15 d after admission)	Chest CT plain scan: The lung window shows multiple mixed-density masses in both lungs, with the margin lobulation and spiculation signs similar to the previous examination. The largest one is located in the posterior basal segment of the left lower lobe, ~6.0 cm × 2.9 cm in size, with CT values ranging from 22.5 to 42.6 HU. A gas-containing cavity within the lesion in the anterior segment of the left upper lobe has slightly increased in size compared to before, and air accumulation is noted within a nodule in the anterior basal segment of the left lower lobe near the oblique fissure. A cystic lucency about 0.9 cm in the lateral segment of the right middle lobe and a few streaky shadows in the right middle lobe are also observed. The mediastinal window shows scattered mildly enlarged lymph nodes in the mediastinum and thickening of the posterior parietal pleura on both sides. Conclusion: Compared with the film on July 3, 2024: Multiple mixed-density masses in both lungs, air accumulation within the nodules in the anterior segment of the left upper lobe and the anterior basal segment of the left lower lobe. Considering the medical history, rheumatoid nodules are suspected. The remaining manifestations are similar to the previous examination
September 3, 2024 (2 mo after discharge)	Chest CT plain scan: Pulmonary window shows: Multiple mixed-density masses in both lungs, with edge features similar to the previous examination. The largest one is located in the outer-posterior basal segment of the left lower lobe, ~4.4 cm × 2.0 cm in size, with CT values of about 28–39 HU. The air-containing cavity in the anterior segment of the left upper lobe has significantly reduced in size compared to before. The air accumulation within the nodule in the anterior basal segment of the right lower lobe near the oblique fissure has been absorbed. A new air-containing nodule is seen in the medial segment of the right middle lobe. A cystic lucency about 0.9 cm in the lateral segment of the right middle lobe. A few linear opacities in the right middle lobe. Mediastinal window shows: Scattered mildly enlarged lymph nodes in the mediastinum; thickening of the posterior costal pleura on both sides. Conclusion: Compared with the film on July 18, 2024: The range of multiple mixed-density masses in both lungs has decreased compared to before; the air-containing lesion in the left upper lobe has reduced in size; the air accumulation in the right lower lobe has been absorbed, and a new air-containing nodule is seen in the right middle lobe, considered as rheumatoid nodules
October 8, 2024 (3 mo after discharge)	Chest CT plain scan: Pulmonary window shows: Multiple mixed-density masses in both lungs, with edge features similar to those in the previous examination. The largest one is located in the posterior basal segment of the left lower lobe, ~3.5 cm × 1.9 cm in size, with CT values of about 28–39 HU. The fluid in the air-containing cystic lesion in the middle lobe of the right lung has been absorbed, and the wall has thinned. The nodule in the horizontal fissure of the right lung has shrunk, and new air accumulation is observed within it. A cystic lucency about 0.9 cm in the lateral segment of the middle lobe of the right lung. A few linear shadows in the middle lobe of the right lung. Mediastinal window shows: Scattered mildly enlarged lymph nodes in the mediastinum; thickening of the lower dorsal pleura on both sides. Conclusion: Compared with the film of 2024-09-03: The multiple mixed-density masses in both lungs have further shrunk; the wall of the air-containing lesion in the middle lobe of the right lung has thinned; the nodule in the horizontal fissure of the right lung has shrunk and air accumulation is present within it, suggesting a rheumatoid nodule.
January 18, 2025 (6 mo after discharge)	Chest CT plain scan: Pulmonary window shows: The bronchovascular bundles in both lungs are naturally distributed. Multiple mixed-density masses are seen in both lungs. The margins of some lesions still show shallow lobulation and spiculation. The largest one is located in the lateral segment of the middle lobe of the right lung, measuring ~3.5 cm × 2.5 cm, with CT values of about 17–32 HU. Some lesions have cavities, with the largest cavity located within the nodule in the medial segment of the middle lobe of the right lung. Mediastinal window shows: No mediastinal deviation, normal morphology of the heart and major blood vessels, and scattered mildly enlarged lymph nodes in the mediastinum. Thickening of the posterior parietal pleura on both sides. Conclusion: Compared with the film of 2024-10-08: Some lesions have decreased in size, some have increased in size, some new cavities have formed, and the walls of some cavities have thickened or are not displayed
April 19, 2025 (9 mo after discharge)	Chest CT plain scan: Pulmonary window shows: The bronchovascular bundles in both lungs are naturally distributed. Multiple mixed-density masses are seen in both lungs. The margins of some lesions still show lobulation and spiculation. The largest one is located in the anterior segment of the right upper lobe, measuring ~2.3 cm × 1.0 cm, with a CT value of about 9 HU. Some new lesions are found in the anterior segment of the right upper lobe, the lateral segment of the right middle lobe, and the posterior basal segment of the left lower lobe. Some lesions have newly developed cavities or changes in the cavities. A few linear shadows are seen in the right middle lobe. Mediastinal window shows: No mediastinal deviation, slightly enlarged cardiac silhouette, normal morphology of major blood vessels, and scattered mildly enlarged lymph nodes in the mediastinum. The posterior paravertebral pleura on both sides is thickened. Conclusion: Compared with the film on January 18, 2025: Some lesions have decreased in size, some are newly developed, and some have newly developed cavities or changes in the cavities. The cardiac silhouette is slightly larger than before
June 28, 2025 (1 yr after discharge)	Chest CT plain scan: Pulmonary window shows: The bronchovascular bundles in both lungs are naturally distributed. Multiple solid patchy and fibrous cord-like shadows are seen in the anterior segment of the upper lobe of both lungs, the middle lobe of the right lung, the posterior basal segment of both lower lobes, the anterior basal segment of the right lower lobe, and the dorsal segment of the left lower lobe, with traction on the adjacent pleura (suggesting morphological changes due to previous lesions); the largest solid patch is located in the medial segment of the middle lobe of the right lung, ~15 mm × 9 mm in size; scattered solid nodules are seen in the dorsal segment of the left lower lobe, with the largest one about 7 mm × 6 mm in size, uniform density, and no cavities; a few cord-like shadows are seen in the lingular segment of the upper lobe of the left lung; the pulmonary hilum is not enlarged. Mediastinal window shows: No mediastinal shift, normal morphology and density of the heart shadow, normal morphology of major blood vessels, and scattered slightly enlarged lymph nodes in the mediastinum (the largest one with a short diameter of about 0.9 cm); thickening of the lower dorsal pleura on both sides. Conclusion: Compared with the film on April 19, 2025: The previously multiple mixed-density masses and nodules have significantly decreased and reduced in size, no cavities are shown, and now mainly consist of multiple solid patchy and fibrous cord-like shadows; the slightly enlarged heart shadow has improved

CT = computed tomography.

**Table 4:** Timeline of the disease course symptoms, medications, key examinations and evolution of chest CT images.

Time	Joint/general symptoms and signs	Key inspection	Treatment (dosage/adjustment)
October 2023 (onset of the disease)	Pain in the metacarpophalangeal joints, wrist joints and knee joints on both sides	–	Untreated
April 3, 2024	Joint symptoms have significantly worsened	A pulmonary nodule of unknown nature was first detected by chest CT	Symptomatic treatment
April 8, 2024		CT-guided lung puncture	Diagnosed with rheumatoid arthritis, the patient was prescribed Ixazomib tablets (25 mg twice daily), methotrexate (10 mg once a week), and oral celecoxib
July 1, 2024	The symptoms were not significantly relieved, so the patient came to our hospital for treatment	Multiple nodules are found in the lungs, and some have significantly enlarged compared with those in the previous hospital	Admit to hospital for treatment
July 3, 2024		The morphology and margin characteristics of the pulmonary nodules are similar to the previous ones. On enhanced scanning, no enhancement is seen in the center, while the margins are significantly enhanced	Continue oral administration of aramidex tablets (25 mg twice daily), MTX (10 mg once a week), celecoxib, and symptomatic treatment
July 9, 2024		Ultrasound-guided lung biopsy	Continue the above-mentioned treatment
July 18, 2024		Bronchoscopic lung biopsy under ultrasound guidance and air accumulation within the nodules in the upper lobe and the anterior basal segment of the lower lobe of the left lung as shown by CT	Considering rheumatoid pulmonary nodules, prednisone tablets 30 mg once daily were added for oral administration
September 3, 2024		CT shows that the range of multiple mixed-density masses in both lungs has decreased compared to before; the air-containing lesion in the upper lobe of the left lung has shrunk; the pneumatocele in the lower lobe of the right lung has absorbed, and a new air-containing nodule is seen in the middle lobe of the right lung	Continue the above-mentioned treatment
October 8, 2024		Mixed density masses in both lungs have further shrunk; the wall of the air-containing lesion in the right middle lobe has thinned; the nodule in the horizontal fissure of the right lung has shrunk and air accumulation has occurred	Continue the above-mentioned treatment
January 18, 2025	Joint symptoms have improved compared to before	Some lesions have shrunk compared to before, some have enlarged, some new cavities have formed, and the walls of some cavities have thickened or are not shown	Discontinue MTX and switch to leflunomide 20 mg/d orally in combination with tocilizumab 8 mg/kg intravenous infusion every 4 weeks. Gradually reduce prednisone to 10 mg/d for maintenance
April 19, 2025		Some lesions have shrunk compared to before, some are newly developed, and some new cavities or changes in existing cavities have been observed	Continue the above-mentioned treatment
June 28, 2025	Joint symptoms were significantly relieved	The previously multiple mixed-density masses and nodules have significantly shrunk and decreased. No cavities are shown. Currently, multiple solid patchy and fibrous cord-like shadows are predominant	Discontinue tocilizumab and continue leflunomide 20 mg/d orally and prednisone 10 mg/d

CT = computed tomography, MTX = methotrexate.

## 3. Discussion

### 3.1. Risk factors and clinical characteristics of pulmonary rheumatoid nodules

Pulmonary rheumatoid nodules (also known as necrotic nodules) are a relatively rare form of lung damage in patients with RA. The prevalence of pulmonary rheumatoid nodules ranges from <0.4% in radiological studies to 32% in lung biopsies of RA patients.^[4]^ This condition is more common in middle-aged or older male smokers or in patients with a long history of RA or those with subcutaneous rheumatoid nodules, but it can also occur in nonsmokers and relatively younger patients. The lesions often appear after RA, but in a few cases, pulmonary rheumatoid nodules may precede arthritis symptoms.^[5]^ Therefore, epidemiological features cannot be used as a basis for exclusion. The pathogenesis of RA-associated lung damage is not yet clear, and the mechanism of its occurrence has not been fully elucidated. It may be related to immune complex deposition, vasculitis-like changes, and focal necrotizing inflammatory responses.^[6]^ Due to persistent vasculitis with ischemic necrosis, about one-third of the nodules can develop cavities.^[7]^ The formation of rheumatoid nodules initially presents as small vasculitis, which gradually evolves into chronic inflammatory granulomas. The typical histological feature of rheumatoid pulmonary nodules is central fibrinoid necrosis surrounded by a palisade of histiocytes, with granulation tissue composed of lymphocytes, plasma cells, and fibroblasts on the outermost layer.^[8]^ On chest imaging, they are manifested as single or multiple, round or irregular ring-shaped nodules, with multiple nodules being more common. The diameters range from 0.5 to 7 cm, and the edges of the nodules are either rough or smooth. They are often located at the periphery of the lungs and near the pleura. About half of the nodules show cavities, mostly thick-walled, which may be a reaction of the surrounding lung parenchyma.^[9]^ RA can present with pleural effusion, hemoptysis, pneumothorax, and bronchopleural fistula. Calcification of rheumatoid nodules is rare, and some patients may have interstitial lung disease, which may be accompanied by mediastinal and hilar lymph node enlargement. Lesions may increase in size, spontaneously regress, or remain unchanged with the progression of the disease.^[10]^ In patients receiving immunosuppressive therapy, it is often difficult to distinguish them from infections or tumors. For example, in RA patients treated with anti-tumor necrosis factor inhibitors, tuberculosis or invasive fungal infections are more common, and their chest imaging manifestations may be similar to those of cavitary or non-cavitary pulmonary rheumatoid nodules.^[11]^ Therefore, if RA patients have nodules of varying sizes on chest CT, the possibility of concurrent pulmonary rheumatoid nodules should be highly suspected. After further examination to rule out the possibility of neoplasms or infections, a basic diagnosis can be made. Generally, actively controlling the condition of RA may lead to the regression of rheumatoid nodules. The clinical significance of pulmonary nodules lies in their potential indication of RA disease activity, and larger nodules or cavitary nodules carry the risk of secondary infection.^[12]^ They are usually associated with subcutaneous nodules and are histopathologically similar to them. For pulmonary rheumatoid nodules, they are generally asymptomatic and usually do not require special treatment.^[13]^ However, if they cause hemoptysis, pleural effusion, spontaneous pneumothorax, or bronchopleural fistula, active treatment is necessary.^[14]^

### 3.2. Differential diagnosis of rheumatoid nodules in the lungs

The imaging findings in this case showed multiple nodules and masses with cavities mainly located subpleurally/peripherally in both lungs. The pathology initially suggested necrosis and inflammatory cell infiltration, so the diagnostic approach initially included the “necrotizing/granulomatous nodules” spectrum, with a focus on differentiating from tuberculosis, fungal infections, sarcoidosis, and malignant lung tumors/lymphomas. Regarding infection, infectious cavitary nodules are more commonly associated with progressive consolidation, fluid levels, significant surrounding inflammatory exudation, and a synchronous rebound of symptoms and inflammatory markers. However, in this patient, TB-DNA, T-SPOT/IGRA, and acid-fast staining were all negative, fungal smear/culture and serum G/GM tests were negative, and no pathogenic sequences were detected in BALF NGS. Additionally, the patient lacked persistent fever, night sweats, and other signs of tuberculosis intoxication. Histologically, there was no evidence of typical infectious granulomas. In terms of tumors, the patient’s tumor markers were negative, and multiple lung and mediastinal lymph node biopsies did not show atypical cells or definite tumor structures (see Table 2). The imaging follow-up showed a “waxing-waning” pattern rather than progressive growth (see Table 3, Fig. 1), reducing the likelihood of a malignant tumor. Sarcoidosis typically requires the pathological confirmation of non-caseatingepithelioid granulomas and a typical imaging distribution. In this case, the mediastinal lymph node biopsy was dominated by lymphocytes, and the lung tissue showed fibrinoid necrosis and histiocyte infiltration, lacking the typical non-caseating granuloma structure, providing insufficient evidence to support sarcoidosis. Additionally, the dynamic changes of cavitary nodules also need to be differentiated from pulmonary infarction/thrombosis and vasculitic nodules. This patient was ANCA negative, and the pathology did not show definite necrotizing vasculitis or inflammatory destruction of the vessel wall. The imaging distribution was more of multiple subpleural nodules rather than typical infarction wedge-shaped lesions. Although the d-dimer was high upon admission, no thrombosis was found in the bilateral lower extremity deep vein color Doppler ultrasound. Therefore, the current diagnosis is more in favor of the rheumatoid nodule spectrum, but long-term imaging follow-up and vigilance for secondary infection of the cavities are still necessary.

Although the diagnosis process of this case involved ruling out multiple diseases, the final diagnosis was not solely based on the “exclusion method” or “treatment response,” but was established on multiple supportive evidences (see Fig. 2 for the specific diagnostic process). Firstly, the histopathological finding of “fibrinoid necrosis” is a typical feature of rheumatoid necrotizing nodules, differentiating it from the caseous necrosis of tuberculosis or the atypical cell proliferation of tumors. Notably, the lung and mediastinal lymph node biopsies in this case did not show the typical “palisading granuloma” structure, where histiocytes are arranged in a palisade around the central necrotic area, which is a classic pathological feature of rheumatoid necrotizing nodules. Nevertheless, the central fibrinoid necrosis and surrounding lymphocyte and histiocyte infiltration observed in the pathology still conform to the basic pathological spectrum of rheumatoid nodules. Due to the limited biopsy sampling (such as only obtaining the necrotic area or the surrounding inflammatory area), the detection rate of the typical palisading structure is not high, and some cases may present as “atypical” or “incomplete” forms. Secondly, the imaging findings of multiple, subpleural, and cavitary nodules are consistent with the typical distribution pattern of rheumatoid nodules reported in the literature. Thirdly, the patient’s serum RF and anti-CCP antibodies were significantly elevated, indicating highly active RA, and pulmonary nodules are often associated with high-titer autoantibodies. Finally, the patient responded well to glucocorticoids and disease-modifying antirheumatic drugs (DMARDs), which is rare in infectious or malignant lesions but common in autoimmune granulomas. Considering the RA background (high-titer RF/anti-CCP), the typical distribution of multiple subpleural cavitary nodules and their dynamic changes during follow-up, as well as the pathological manifestations dominated by necrosis/histiocytes and negative acid-fast staining (see Table 2), the diagnosis of rheumatoid pulmonary nodules is ultimately more supported; however, given the limitations of biopsy sampling, long-term imaging follow-up and vigilance for secondary infection of the cavities are still necessary.

### 3.3. Treatment and prognosis of pulmonary rheumatoid nodules

The treatment of RA-related lung damage is mainly based on non-steroidal anti-inflammatory drugs and DMARDs, with the addition of glucocorticoids. The initial treatment dose of glucocorticoids is 0.5 to 1.0 mg/kg/d. After a comprehensive assessment of the patient’s clinical symptoms and signs, laboratory tests, chest imaging manifestations, and pulmonary function shows improvement, the dose of glucocorticoids can be gradually reduced or maintained at <10 mg/d for long-term low-dose oral treatment. In this case, the patient was treated with glucocorticoids (prednisone 30 mg/d) combined with DMARDs to control active RA and lung inflammation. Short-term use of glucocorticoids can effectively relieve symptoms and slow the progression of joint damage.^[15]^ Therefore, preventive measures (calcium supplementation, gastric mucosa protection) were also taken during the treatment to reduce the risk of osteoporosis and gastrointestinal adverse reactions.^[16]^

It should be noted that traditional DMARDs, which are effective in improving joint damage symptoms and controlling the progression of RA, may also cause drug-related lung damage. MTX and LEF are first-line drugs for the treatment of RA, but they are also the most common drugs causing lung damage. However, the mechanism by which DMARDs cause lung damage in RA patients is still unclear. Some scholars have proposed that although MTX does not directly aggravate lung damage, it can increase the incidence of pneumonia in RA patients with existing lung damage. Therefore, it is not recommended for RA patients with existing lung damage to use this drug.^[17]^

There is some controversy regarding the continued use of MTX in the treatment plan for this patient. The reasons are as follows: first, the pulmonary nodules in this patient were discovered in a chest CT at an outside hospital before the initiation of MTX, thus the appearance of the nodules does not conform to the typical pattern of new onset after MTX exposure; however, it should be acknowledged that MTX may affect the subsequent evolution of the nodules, so follow-up observation is still necessary. Second, MTX-related pneumonia usually presents with acute/subacute dyspnea, fever, etc, and the imaging is mainly characterized by diffuse ground-glass opacity/interstitial changes, often accompanied by a decline in diffusion function; while in this case, the imaging is mainly characterized by multiple subpleural nodules/masses and cavity formation, and multiple etiological examinations and pathology do not support infection or tumor, which is more consistent with the spectrum of necrotic rheumatoid nodules. Third, regarding the possibility of “accelerated sarcoidosis by MTX,” this condition is often manifested as a rapid increase in the number of nodules in a short period, commonly in subcutaneous nodules, and can also involve the lungs. Although there were asynchronous fluctuations in the size of the nodules and the formation of cavities during the follow-up of this case (waxing–waning), the overall trend was gradually absorbed after the treatment with glucocorticoids combined with DMARDs. In addition, during the follow-up of this case, there was an asynchronous evolution of “partial lesions shrinking and absorbing, new lesions developing, and some solid nodules transforming into cavities and then reabsorbing” (Table 3). This kind of manifestation is not uncommon in the pathological changes of pulmonary rheumatoid nodules: Firstly, different nodules may be at different pathological stages (inflammatory infiltration - central fibrinoid necrosis - necrotic liquefaction/formation of cavities - fibrous cord formation/scarring absorption), thus presenting a coexistence of retraction and cavitation in the imaging at the same time point. Secondly, there may be a certain time lag in the imaging changes of the nodules. Even after discontinuation (or adjustment) of MTX, existing nodules may continue to undergo necrosis and cavity formation for a period of time; at the same time, during the immunosuppressive treatment process, if the overall inflammatory activity of RA still fluctuates (the patient in this case had a significantly elevated inflammatory response and high titers of RF/anti-CCP at admission), new nodules may also occur in stages. Therefore, there is insufficient evidence that MTX induces or significantly accelerates the progression of nodules. Fourth, in the early treatment stage, due to the limited experience of the attending physician in the standard treatment strategy for pulmonary rheumatoid nodules, and the patient’s joint symptoms and inflammatory activity were high, MTX was used as one of the basic DMARDs to quickly control the activity of RA and was closely monitored. With the improvement of multidisciplinary discussions and follow-up, the patient’s treatment plan was adjusted according to the opinion of the rheumatology and immunology specialist, and MTX was discontinued, and the treatment was adjusted to LEF 20 mg/d po combined with tocilizumab 8 mg/kg ivgtt q4w. At the same time, an imaging follow-up (CT every 3–6 months) and infection monitoring (sputum culture/bronchoscopy if necessary) plan was formulated to reduce the risk of secondary infection of cavity nodules and drug adverse reactions. Based on the comprehensive judgment of the causal assessment of adverse reactions: the pulmonary nodules in this case appeared earlier than MTX exposure, and there was no evidence of rapid reversal after drug withdrawal or recurrence upon rechallenge; combined with pathology and typical distribution imaging, it is more consistent with the spectrum of RA-related necrotic nodules. Therefore, there is insufficient evidence that MTX induces or significantly accelerates sarcoidosis, but during the treatment process, the risk of MTX-related lung toxicity and opportunistic infections should still be vigilant and monitored.

In this case, the patient demonstrated a significant and sustained therapeutic response to the treatment regimen of prednisone combined with DMARDs, providing important insights for clinical practice. After the initiation of treatment, the patient’s joint pain and cough symptoms were rapidly alleviated, and their quality of life was significantly improved, suggesting that RA with high disease activity and associated pulmonary inflammation are highly sensitive to immunosuppressive therapy. A 1-year follow-up chest CT scan showed gradual absorption of pulmonary nodules, especially the radiological improvement of cavitary nodules, confirming that such lesions have a certain degree of reversibility. However, long-term imaging follow-up is still necessary to monitor the risk of recurrence and secondary infection. Based on the characteristics of cavitary nodules, in this case, the follow-up focused on the risk of secondary infection and complications: once fever, rebound of inflammatory indicators, thickening of the cavity wall/ fluid level or hemoptysis occurred, sputum/ lavage fluid etiology should be reexamined as a priority and whether bronchoscopic sampling is needed should be evaluated, while shortening the interval of imaging follow-up. In addition, the patient had high-titer RF and anti-CCP antibodies before treatment. These serological markers are usually associated with more severe extra-articular manifestations^[18]^; although the changes in antibody titers after treatment were not provided in the article, their dynamic monitoring has potential value in evaluating treatment response and predicting the risk of recurrence. RA often recurs and remits. Although some patients have mild conditions, others may suffer from a disease that seriously affects their quality of life. It is important to pay attention to and manage the prognosis of patients. Poor prognosis outcomes are often seen in patients with high-titer autoantibodies, HLA-DRB1 genotype, onset age <30 years, multiple joint involvement, female gender, and extra-articular involvement at the time of presentation.^[19]^

## 4. Conclusion

The case reported in this article has certain particularities. Firstly, the patient is a 33-year-old young female, while rheumatoid nodules in the lungs are more commonly seen in literature among male smokers with a longer disease history and positive serology. This indicates that clinicians should not rely solely on typical epidemiological characteristics to make judgments. For any RA patient presenting with unexplained pulmonary nodules, pulmonary rheumatoid nodules should be included in the differential diagnosis. Secondly, the diagnostic process of this case was tortuous, highlighting the complexity of excluding common causes such as infection and tumors, and emphasizing the importance of multidisciplinary collaboration and repeated biopsies when necessary. This case suggests that for RA patients with pulmonary nodules, a “rule-out - support” dual-track strategy should be adopted: while excluding common causes such as infection and tumors, actively seek evidence supporting rheumatoid lung damage (such as typical imaging, pathological features, serological markers, and treatment responses) to improve diagnostic accuracy. Pulmonary damage can exacerbate the severity of RA and even directly lead to death. In clinical practice, due to the fact that early pulmonary damage in RA patients often does not present symptoms and the late clinical manifestations are not specific, it is easily confused with primary lung diseases, thus it is easily overlooked in clinical diagnosis and treatment. Therefore, it is very important to pay attention to pulmonary damage associated with RA and conduct early intervention based on the risk factors of the disease.

## 5. Limitations

Although the pathological examination in this study found fibrinoid necrosis, the typical “palisade granuloma” structure was not observed, which might be related to the limited sampling of the biopsy, leading to atypical histological manifestations. Additionally, no PET/CT examination was conducted, lacking an assessment of the metabolic activity of the nodules, which affected the comprehensiveness of the differential diagnosis. There is some controversy over the continued use of MTX in the treatment plan, as MTX itself may induce lung injury. Although the pulmonary nodules were discovered before the use of MTX, suggesting that the lung damage is more likely to be caused by RA itself, its potential impact during this process cannot be completely ruled out. Moreover, the patient’s fingertip oxygen saturation was low upon admission, but after hospital treatment, the blood oxygen saturation monitoring was basically normal. The patient refused to undergo blood gas analysis and pulmonary function tests, so this case lacks an assessment of the patient’s lung function. Finally, only 1 year of follow-up has been conducted so far. Although the pulmonary lesions have gradually absorbed, the long-term prognosis and recurrence risk still require longer-term observation

## Author contributions

**Conceptualization:** Weiran Li, Sai Yuan, Xue Wu.

**Formal analysis:** Weiran Li, Sai Yuan.

**Methodology:** Sai Yuan.

**Project administration:** Mao Hua.

**Supervision:** Weiran Li, Sai Yuan.

**Writing – original draft:** Sai Yuan, Weiran Li, Zhongping Wang, Jin Guo, Hao Yang.

**Writing – review & editing:** Mao Hua, Sai Yuan, Weiran Li.
